# Echocardiographic abnormalities and joint hypermobility in Chinese patients with Osteogenesis imperfecta

**DOI:** 10.1186/s13023-024-03089-x

**Published:** 2024-03-12

**Authors:** Yazhao Mei, Yunyi Jiang, Li Shen, Zheying Meng, Zhenlin Zhang, Hao Zhang

**Affiliations:** 1 Shanghai Clinical Research Center of Bone Disease, Department of Osteoporosis and Bone Disease, Shanghai Sixth People’s Hospital Affiliated to Shanghai Jiao Tong University School of Medicine, 200233 Shanghai, China; 2 Clinical Research Center, Shanghai Sixth People’s Hospital Affiliated to Shanghai Jiao Tong University School of Medicine, 200233 Shanghai, China; 3 Department of Ultrasound, Shanghai Sixth People’s Hospital Affiliated to Shanghai Jiao Tong University School of Medicine, 200233 Shanghai, China

**Keywords:** Osteogenesis imperfecta, Type I collagen, Echocardiography, Joint hypermobility

## Abstract

**Background:**

Very little is known about the characteristics of echocardiographic abnormalities and joint hypermobility in Chinese patients with osteogenesis imperfecta (OI). The aim of our study was to investigate the characteristics, prevalence and correlation of echocardiographic abnormalities and joint hypermobility in Chinese patients with OI.

**Methods:**

A cross-sectional comparative study was conducted in pediatric and adult OI patients who were matched in age and sex with healthy controls. Transthoracic echocardiography was performed in all patients and controls, and parameters were indexed for body surface area (BSA). The Beighton score was used to evaluate the degree of joint hypermobility.

**Results:**

A total of 48 patients with OI (25 juveniles and 23 adults) and 129 age- and sex-matched healthy controls (79 juveniles and 50 adults) were studied. Four genes (*COL1A1*, *COL1A2*, *IFITM5*, and *WNT1*) and 39 different mutation loci were identified in our study. Mild valvular regurgitation was the most common cardiac abnormality: mild mitral and tricuspid regurgitation was found in 12% and 36% of pediatric OI patients, respectively; among 23 OI adults, 13% and 17% of patients had mild mitral and tricuspid regurgitation, respectively, and 4% had mild aortic regurgitation. In multiple regression analysis, OI was the key predictor of left atrium diameter (LAD) (β=-3.670, *P* < 0.001) and fractional shortening (FS) (β = 3.005, *P* = 0.037) in juveniles, whereas for adults, OI was a significant predictor of LAD (β=-3.621, *P* < 0.001) and left ventricular mass (LVM) (β = 58.928, *P* < 0.001). The percentages of generalized joint hypermobility in OI juveniles and adults were 56% and 20%, respectively. Additionally, only in the OI juvenile group did the results of the Mann‒Whitney U test show that the degree of joint hypermobility was significantly different between the echocardiographic normal and abnormal groups (*P* = 0.004).

**Conclusions:**

Mild valvular regurgitation was the most common cardiac abnormality in both OI juveniles and adults. Compared with OI adults, OI juveniles had more prevalent and wider joint hypermobility. Echocardiographic abnormalities may imply that the impairment of type I collagen is more serious in OI. Baseline echocardiography should be performed in OI patients as early as possible.

**Supplementary Information:**

The online version contains supplementary material available at 10.1186/s13023-024-03089-x.

## Introduction

Osteogenesis imperfecta (OI) is a clinically and genetically heterogeneous group of hereditary bone disorders [1]. In OI, approximately 85-90% of autosomal dominant disorders are caused by mutations in the *COL1A1* and *COL1A2* genes, which encode the α1(I) and α2(I) chains of type I collagen [2]. Since the first identification of the gene resulting in recessive OI in 2006, over 20 different genes involved in the biosynthesis of type I collagen to cause OI have been reported [3]; therefore, currently, OI is widely accepted as a collagen-related disease [2]. According to data from Europe and the United States, the incidence of OI is approximately 0.3–0.7 per 10,000 births, and the number varies in different regions [4].

The severity of OI ranges from individuals with a few fractures to some with severe skeletal deformities and perinatal lethality [5]. Type I collagen is the major protein component of the extracellular matrix in bone, skin, tendon, heart, and so on; therefore, extra-skeletal manifestations such as blue sclera, dentinogenesis imperfecta, hearing impairment, ligamentous laxity, and cardiovascular abnormalities are also common in patients [4, 6]. Most of the research on OI undertaken to date has focused on the characteristics of bone mineral density (BMD) and fracture rates, as well as related therapies; however, these indicators fail to adequately reflect the patient’s health status and quality of life [7]. A study of the mortality of patients with OI in Denmark has shown that diseases of the circulatory system were the most common cause of death (2.9%) [8]. Type I collagen covers approximately 74% of the collagen content of the mitral valve [9]. The aorta and most arteries contain abundant type III and type I collagen [10]. Preliminary experimental evidence based on OI mouse models has suggested that mutations in *COL1A1* or *COL1A2* will increase the risk of cardiovascular diseases. Previous clinical studies [11–14] have also demonstrated that patients with OI have an increased risk of heart disease compared to healthy controls, and valvular diseases and widened aortic diameter were the most frequently reported disorders. However, very little is known about the clinical characteristics of the cardiovascular system in OI, and limited attention has been given to cardiovascular screening of patients with OI in China.

Joint hypermobility is the range of movement exceeding the normal level considering age, sex, and ethnic background [15]. Ligamentous laxity is the primary cause of hypermobility. The Beighton score, an easy-to-use scoring system to measure joint hypermobility, is currently the most widely used method for assessing the presence of generalized joint hypermobility [16]. The varying degrees of joint hypermobility in different types of EDS, OI and Marfan syndrome form a heterogeneous group of hereditary connective tissue disorders [17]. Joint hypermobility is a common characteristic of patients with OI [18], which may increase the risk of falls and fractures. EDS is characterized by joint hypermobility, soft and hyperextensible skin, and abnormal scar formation after injury [19]. Recently, patients with overlapping phenotypes of OI and EDS have been found to harbor heterozygous pathogenic variants in *COL1A1* or *COL1A2* [20, 21], and Morlino et al. proposed the term “*COL1*-related overlap disorder (C1ROD)” to consider a wide spectrum of bridging phenotypes between OI and EDS [22]. The variants found in C1ROD were mainly located in the N-terminal helical region, whereas some variants were also reported to be located outside the region [23].

Very little is known about the characteristics of structural cardiovascular abnormalities and joint hypermobility in Chinese patients with OI. The primary objective of this cross-sectional study is to investigate the characteristics and prevalence of echocardiographic abnormalities and joint hypermobility in Chinese patients with OI. We also analyzed the association between these two phenotypes in our cohort.

## Materials and methods

### Subjects

This was a single-center cross-sectional study of patients with OI consulted at the Department of Osteoporosis and Bone Disease of Shanghai Sixth People’s Hospital affiliated to Shanghai Jiao Tong University School of Medicine from June 2021 to October 2022. Patients who met all these criteria were included: (1) confirmed with OI-related gene variants, (2) underwent clinical evaluation, (3) underwent echocardiography, and (4) without hypertension or other chronic cardiovascular diseases. Individuals matched for age and sex with no history of cardiovascular disease, hypertension, or diabetes were voluntarily recruited for echocardiography as normal controls. The Ethics Committee of Shanghai Sixth People’s Hospital Affiliated to Shanghai Jiao Tong University School of Medicine approved the study. Written informed consent was obtained from patients or legal guardians of juveniles younger than 18.

### Clinical evaluation

Clinical information, including age, sex, height, weight, fracture history, non-skeletal features (such as blue sclera, dentinogenesis imperfecta, and hearing loss), typical cardiovascular symptoms (such as chest pain, dyspnea, palpitations, and syncope), and medical history were collected. Body surface area (BSA) was calculated using the Mosteller formula [24]. Blood pressure was measured with an OMRON U16 (OMRON HEALTHCARE Co. Ltd.) electronic sphygmomanometer. Hypertension was defined when systolic blood pressure (SBP) ≥ 140 mmHg or diastolic blood pressure (DBP) ≥ 90 mmHg was measured or when the participants were administered antihypertensive medication. In addition, X-ray radiography of the upper and lower extremities and thoracolumbar vertebrae were examined. Patients were typed 1 to 5 according to the *Nosology of Genetic Skeletal Disorders: 2023 Revision* [25].

### Echocardiographic examination

Transthoracic echocardiography was performed with Philips EPIQ 7 C. The echocardiography protocol was performed by two experienced cardiologists at the same time, and the echocardiograms were interpreted by experienced cardiologists. Atrial, ventricular, valvular, and aortic measurements were performed according to the guidelines of the American Society of Echocardiography [26]. Two-dimensional measurements, M-mode measurements, and Doppler ultrasonography were used to measure aorta diameter (AD), left atrium diameter (LAD), interventricular septum thickness (IVST), left ventricular posterior wall thickness (LVPWT), left ventricular end-diastolic diameter (LVEDd), left ventricular end-systolic diameter (LVESd), left ventricular ejection fraction (LVEF), fractional shortening (FS), and valvular regurgitation. Left ventricular mass (LVM) was calculated according to the American Society of Echocardiography’s (ASE)-recommended formula [26]. Echocardiographic parameters can be influenced by BSA, so the measurements were divided by BSA according to the recommendations of the ASE [26]. All detectable regurgitations were recorded and divided into mild, moderate, or severe [27]. Based on the echocardiographic reports, patients were divided into two subgroups: (1) normal and (2) abnormal (including but not limited to valvular regurgitation, atrioventricular enlargement, ventricular septal thickening, and ascending aortic dilatation).

#### Joint Hypermobility Assessment

The presence or absence of joint hypermobility and severity were assessed by the smae experienced physician to minimize variability. The Beighton scoring system was used to evaluate the joint hypermobility severity [16]. A score of four or higher was considered a positive result for generalized joint hypermobility. According to the physical examination and Beighton score, we divided the results of the joint hypermobility assessment into three subgroups: (1) normal (absence of joint hypermobility); (2) peripheral joint hypermobility (mainly hands); and (3) generalized joint hypermobility (Beighton score ≥ 4) [28].

### Detection of gene mutations

Genomic DNA was extracted from the peripheral blood of all patients by standard techniques using a DNA extraction kit (Lifefeng Biotech, Shanghai). Exons and exon‒intron boundaries of *COL1A1*, *COL1A2*, *IFITM5*, and *WNT1* genes were amplified by polymerase chain reaction (PCR) (GenBank accession NO. NC_000017.11, NO. NC_000007.14, NO.NC_000011.10, and NO.NC_000012.12). Primers were designed by Web-based Primer 3 software (https://bioinfo.ut.ee/primer3-0.4.0/). Direct sequencing was performed using BigDye Terminator Cycle Sequencing Ready Reaction Kit, v. 3.1 (Applied Biosystems, California, USA). The products were analyzed with an ABI 3730 sequencer (Foster, CA, United States). SNPs were identified using Polyphred (https://droog.gs.washington.edu/polyphred/). The results absent from the LOVD Database (https://www.lovd.nl/) were regarded as novel variants.

### Statistical analysis

Continuous variables were expressed as the means ± SDs. Total counts and percentages were reported for categorical variables. The chi-square test was used to assess the differences in proportions between OI and controls. Group differences in continuous variables between OI patients and controls were completed by independent-sample t tests. Simple and multiple linear regression models were used to investigate the impacts of possible variables on echocardiographic parameters. The Mann‒Whitney U test was applied to analyze the correlation between cardiovascular abnormalities and joint hypermobility. Two-tailed *P* values less than 0.05 were considered statistically significant. SPSS 26.0 software (SPSS Inc., Chicago, IL) was used for statistical analysis.

## Results

### Baseline characteristics and genotypes of the subjects

In all, 48 patients with OI (25 juveniles and 23 adults) and 129 age- and sex-matched healthy controls (79 juveniles and 50 adults) were studied. Twenty-one patients with OI were reported in our previous study [29]. Baseline characteristics are shown in Table 1. For juveniles, the mean age of OI patients was 11.0 ± 4.4 years when receiving echocardiographic examinations, and 18 (72%) patients were boys; the control group had 42 boys and 37 girls, with a mean age of 12.6 ± 3.5 years. For adults, the mean ages of OI patients and healthy controls were 39.54 ± 12.18 years and 41.00 ± 10.75 years, respectively; there were 6 (26%) and 25 (50%) males in the OI and control groups, respectively. The two OI groups had significantly lower BSA than the control individuals, 1.3 ± 0.5 VS 1.5 ± 0.4 m^2^ in juveniles (*P* = 0.013), and 1.5 ± 0.2 VS 1.7 ± 0.2 m^2^ in adults (*P* < 0.001), respectively. There was no difference in SBP and DBP between the OI groups and controls. Among the 48 patients with OI, only two adult patients experienced cardiac disease-related symptoms or treatments: one patient underwent aortic valve prosthetic replacement when she was 25 years old because of a congenital aortic valve defect, and the other had episodic palpitation.

**Table 1:** Baseline characteristics of osteogenesis imperfecta patients and controls

	OI-Juveniles	Control-Juveniles	*P *	OI-Adults	Control-Adults	*P*
Number of patients	25	79	/	23	50	/
Age (± SD), year	11.0 ± 4.4	12.6 ± 3.5	0.051	39.54 ± 12.18	41.00 ± 10.75	0.608
Sex, male/female	18/7	42/37	0.097	6/17	25/25	0.055
Height (± SD), cm	138.2 ± 26.2	156.7 ± 18.5	**0.003**	153.3 ± 9.9	166.7 ± 8.0	**<0.001**
Weight (± SD), kg	43.2 ± 24.3	51.9 ± 18.9	0.064	52.0 ± 10.2	65.7 ± 14.0	**<0.001**
BSA (± SD), m^2^	1.3 ± 0.5	1.5 ± 0.4	**0.013**	1.5 ± 0.2	1.7 ± 0.2	**<0.001**
SBP, mmHg	109.2 ± 18.3	113.2 ± 9.8	0.348	115.8 ± 12.2	118.6 ± 10.9	0.352
DBP, mmHg	70.1 ± 8.1	71.3 ± 8.1	0.700	75.8 ± 8.1	76.2 ± 8.1	0.855
Lumbar spine BMD(± SD), g/cm^2^	0.53 ± 0.19	/	/	0.81 ± 0.14	/	/
Lumbar spine BMD Z score (± SD)	-1.80 ± 1.87	/	/	-2.20 ± 1.07	/	/
Femoral neck BMD(± SD), g/cm^2^	0.52 ± 0.19	/	/	0.71 ± 0.13	/	/
Femoral neck BMD Z score (± SD)	-3.0 ± 1.48	/	/	-1.34 ± 0.96	/	/
Blue sclera, n (%)	16 (64%)	/	/	20 (87%)	/	/
Dentinogenesis imperfecta, n (%)	8 (32%)	/	/	8 (35%)	/	/
Hearing loss, n (%)	1 (4%)	/	/	11 (48%)	/	/
Scoliosis, n (%)	2 (8%)	/	/	3 (13%)	/	/
Cardiac diseases related symptoms/treatment, n (%)	0	/	/	2 (9%)	/	/
Type 1	15 (60%)	/	/	20 (87%)	/	/
Type 3	1 (4%)	/	/	0	/	/
Type 4 Type 5	8 (32%) 1 (4%)	/ /	/ /	2 (9%) 1 (4%)	/ /	/ /

*Abbreviations* OI: osteogenesis imperfecta; BSA: body surface area; SBP: systolic blood pressure; DBP: diastolic blood pressure. Bold *P* values indicate significance.

Skeletal and extra-skeletal manifestations of patients with OI are displayed in Table 1. Two (8%) juveniles and 3 (13%) adults with OI had scoliosis. Both lumbar spine and femoral neck BMD values were low in juvenile and adult patients with OI: the lumbar spine BMD Z score was − 1.80 ± 1.87 in juveniles and − 2.20 ± 1.07 in adults, and the femoral neck BMD Z score was − 3.00 ± 1.48 in juveniles and − 1.34 ± 0.96 in adults. Blue sclera was a very common extra-skeletal manifestation in the two OI groups, and hearing loss was much more frequent in adults than in juveniles with OI. According to the classification, there were 15 juvenile patients with OI type 1, 1 with type 3, 8 with type 4, and 1 with type 5; in adults, type 1 included 20 patients, type 4 contained 2, and type 5 involved 1.

A total of four genes (*COL1A1*, *COL1A2*, *IFITM5*, and *WNT1*) and 39 different mutation loci were identified in our study. Thirty patients carried *COL1A1* gene mutations, including 11 nonsense mutations, 9 missense mutations, 8 frameshift mutations, one splice mutation, and one gross deletion (chr17:48262862–48,268,851, approximately 5.99 kb). Sixteen patients harbored *COL1A2* gene mutations, including 14 missense mutations and 2 splice mutations. A pathogenic variant in *IFITM5* was detected in two patients, and one boy carried *WNT1* compound heterozygous variants. Three variants (c.1865dupC and a gross deletion in *COL1A1*, c.1649G > C in *COL1A2*) absent in the LOVD Database (https://www.lovd.nl/) were novel variants.

### Echocardiographic measurements of the subjects

Mild mitral regurgitation was found in 12% and mild tricuspid regurgitation in 36% of patients in the OI juvenile group. Among 23 OI adults, 13% and 17% of patients had mild mitral and tricuspid regurgitation, respectively, 4% had mild aortic regurgitation, 4% had left atrium enlargement and increased pulmonary artery pressure, respectively, and only one (4%) had experienced aortic valve replacement. Detailed information on the patients mentioned above is displayed in Supplementary Table 1.

Echocardiographic parameters in OI patients and controls and their comparisons are shown in Table 2. There was no difference between the OI and control groups regarding echocardiographic parameters, except for AD and LVM, which were much larger in control adults than in OI adults. However, when corrected for BSA, AD, LAD, IVST, LVEDd, LVESd, and FS became significantly higher in the OI juveniles than in the control juveniles. AD, LAD, IVST, LVPWT, LVEDd, LVESd, LVEF, and FS became significantly larger in the OI adults than in the control adults when corrected for BSA.

**Table 2:** Echocardiographic parameters in osteogenesis imperfecta patients and controls

	OI- Juveniles	Control- Juveniles	*P*	OI-Adults	Control-Adults	*P*
AD (cm)	21.44 ± 4.49	21.94 ± 2.82	0.606	26.03 ± 2.40	27.28 ± 2.30	**0.038**
AD/BSA	18.47 ± 4.56	15.36 ± 2.98	**0.003**	17.72 ± 2.25	15.85 ± 1.62	**0.001**
LAD (cm)	28.96 ± 6.89	28.28 ± 4.64	0.647	31.78 ± 3.20	31.26 ± 4.27	0.603
LAD/BSA	24.63 ± 5.41	19.69 ± 3.73	**<0.001**	21.63 ± 2.88	18.09 ± 2.17	**<0.001**
IVST (cm)	6.68 ± 1.41	7.20 ± 1.20	0.072	8.13 ± 1.32	8.24 ± 1.17	0.723
IVST/BSA	5.81 ± 1.66	5.02 ± 1.01	**0.033**	5.51 ± 0.92	4.79 ± 0.76	**0.001**
LVPWT (cm)	6.52 ± 1.44	7.13 ± 1.10	0.073	7.83 ± 1.14	7.96 ± 0.99	0.627
LVPWT/BSA	5.65 ± 1.59	4.99 ± 1.07	0.075	5.28 ± 0.93	4.62 ± 0.63	**0.006**
LVEDd (cm)	41.42 ± 7.83	42.59 ± 5.24	0.495	43.30 ± 3.55	44.44 ± 3.55	0.208
LVEDd/BSA	35.83 ± 9.22	29.83 ± 5.79	**0.005**	29.49 ± 3.66	25.84 ± 2.68	**<0.001**
LVESd (cm)	26.92 ± 4.99	27.51 ± 3.75	0.535	28.04 ± 2.64	28.62 ± 2.72	0.398
LVESd/BSA	23.32 ± 6.17	19.26 ± 3.91	**0.005**	19.10 ± 2.57	16.64 ± 1.93	**<0.001**
LVEF	62.91 ± 9.45	64.96 ± 3.68	0.910	64.43 ± 2.93	64.96 ± 3.83	0.571
LVEF/BSA	33.95 ± 15.95	47.01 ± 15.15	0.318	43.55 ± 5.02	37.98 ± 5.23	**<0.001**
FS	35.52 ± 3.49	35.43 ± 2.95	0.910	35.27 ± 2.42	35.46 ± 3.19	0.796
FS/BSA	33.95 ± 15.95	25.63 ± 8.28	**0.024**	24.06 ± 3.08	20.73 ± 3.15	**<0.001**
LVM (g)	84.93 ± 44.33	93.18 ± 32.47	0.413	102.04 ± 30.81	188.35 ± 39.06	**<0.001**
LVM/BSA	42.47 ± 22.16	46.59 ± 16.24	0.413	68.71 ± 19.61	108.32 ± 17.41	**<0.001**

*Abbreviations* OI: osteogenesis imperfecta; BSA: body surface area; AD: aorta diameter; LAD: left atrium diameter; IVST: interventricular septum thickness; LVPWT: left ventricular posterior wall thickness; LVEDd: left ventricular end-diastolic diameter; LVESd: left ventricular end-systolic diameter; LVEF: left ventricular ejection fraction; FS: fractional shortening; LVM: Left ventricular mass. Bold *P* values indicate significance.

**Table 3:** Simple regression analysis of echocardiographic parameters

	Dependent variable																	
Independent variable	AD		LAD		IVST		LVPWT		LVEDd		LVESd		LVEF		FS		LVM	
	β	*P*	β	*P*	β	*P*	β	*P*	β	*P*	β	*P*	β	*P*	β	*P*	β	*P*
**A. In the juvenile group**																		
Age	0.669	**<0.001**	0.838	**<0.001**	0.225	**<0.001**	0.231	**<0.001**	1.128	**<0.001**	0.714	**<0.001**	-0.081	0.316	0.012	0.932	6.905	**<0.001**
Sex	-2.283	**<0.001**	-2.067	**0.047**	-0.448	0.074	-0.462	0.055	-4.765	**<0.001**	-3.144	**<0.001**	0.526	0.393	1.719	0.121	-25.242	**<0.001**
BSA	6.616	**<0.001**	10.006	**<0.001**	2.241	**<0.001**	2.150	**<0.001**	12.425	**<0.001**	8.030	**<0.001**	-0.938	0.220	-1.246	0.370	73.623	**<0.001**
SBP	0.164	**<0.001**	0.203	**<0.001**	0.051	**<0.001**	0.039	**<0.001**	0.237	**<0.001**	0.129	**<0.001**	0.026	0.322	0.071	0.134	1.396	**<0.001**
DBP	0.161	**<0.001**	0.209	**<0.001**	0.045	**0.001**	0.041	**0.001**	0.220	**<0.001**	0.118	**0.006**	0.036	0.274	0.083	0.168	1.351	**<0.001**
OI	0.497	0.512	-0.682	0.574	0.523	0.072	0.605	**0.034**	1.178	0.396	0.590	0.535	-0.091	0.900	2.049	0.119	8.252	0.328
**B. In the adult group**																		
Age	0.062	**<0.001**	0.110	**0.008**	0.009	0.480	0.007	0.510	0.003	0.940	-0.035	0.213	0.076	**0.047**	0.069	**0.027**	0.302	0.602
Sex	-1.261	**0.025**	-0.327	0.729	-0.540	0.060	-0.426	**0.083**	-2.436	**0.003**	-1.649	**0.009**	0.452	0.601	0.356	0.614	-45.668	**<0.001**
BSA	5.201	**<0.001**	7.107	**<0.001**	1.510	**0.013**	1.412	**0.008**	7.217	**<0.001**	4.406	**0.001**	-0.128	0.946	-0.051	0.973	169.534	**<0.001**
SBP	0.034	0.185	0.002	0.960	0.005	0.697	0.007	0.516	0.015	0.705	-0.012	0.678	0.047	0.229	0.039	0.223	0.444	0.435
DBP	0.031	0.385	0.053	0.382	0.009	0.635	0.017	0.269	0.065	0.231	0.024	0.554	0.003	0.952	0.004	0.930	0.689	0.386
OI	1.245	**0.038**	-0.523	0.603	0.110	0.723	0.131	0.627	1.136	0.208	0.577	0.398	0.531	0.571	0.195	0.796	86.304	**<0.001**

*Abbreviations* OI: osteogenesis imperfecta; BSA: body surface area; SBP: systolic blood pressure; DBP: diastolic blood pressure; AD: aorta diameter; LAD: left atrium diameter; IVST: interventricular septum thickness; LVPWT: left ventricular posterior wall thickness; LVEDd: left ventricular end-diastolic diameter; LVESd: left ventricular end-systolic diameter; LVEF: left ventricular ejection fraction; FS: fractional shortening; LVM: Left ventricular mass. Bold *P* values indicate significance.

**Table 4:** Multiple regression analysis of echocardiographic parameters

	Dependent variable																	
Independent variable	AD		LAD		IVST		LVPWT		LVEDd		LVESd		LVEF		FS		LVM	
	β	*P*	β	*P*	β	*P*	β	*P*	β	*P*	β	*P*	β	*P*	β	*P*	β	*P*
**A. In the juvenile group**																		
Age	0.372	**<0.001**	-0.069	0.660	0.100	**0.026**	0.149	**<0.001**	0.161	0.289	0.089	0.446	-0.071	0.633	0.276	0.291	1.715	0.072
Sex	-0.774	0.051	0.397	0.570	-0.009	0.965	-0.033	0.849	-2.222	**0.001**	-1.471	**0.005**	0.310	0.640	1.342	0.247	-10.476	**0.014**
BSA	2.394	**0.008**	10.987	**<0.001**	1.277	**0.005**	0.894	**0.026**	10.730	**<0.001**	7.576	**<0.001**	-1.117	0.453	-5.276	**0.046**	57.899	**<0.001**
SBP	0.040	0.094	0.011	0.796	0.011	0.342	-0.008	0.442	-0.009	0.818	-0.035	0.258	0.044	0.276	0.096	0.168	-0.100	0.691
DBP	0.036	0.189	0.039	0.424	0.001	0.944	0.013	0.276	0.025	0.599	0.010	0.787	0.025	0.589	0.044	0.583	0.235	0.420
OI	-0.678	0.159	-3.670	**<0.001**	-0.043	0.858	0.103	0.633	-1.181	0.156	-1.016	0.113	0.494	0.549	3.005	**0.037**	-8.018	0.123
**B. In the adult group**																		
Age	0.061	**0.006**	0.097	**0.004**	0.011	0.380	0.008	0.488	0.008	0.821	-0.032	0.240	0.074	0.067	0.067	**0.038**	0.167	**0.014**
Sex	-0.027	0.965	2.498	**0.009**	-0.204	0.574	-0.076	0.805	-0.569	0.565	-0.362	0.639	0.124	0.910	0.103	0.910	-4.965	0.591
BSA	5.347	**0.001**	14.027	**<0.001**	1.615	0.065	1.569	**0.036**	7.383	**0.002**	4.600	**0.015**	-0.631	0.811	-0.102	0.962	102.379	**<0.001**
OI	-0.205	0.730	-3.621	**<0.001**	-0.365	0.298	-0.280	0.352	-0.883	0.355	-0.629	0.398	0.532	0.620	0.147	0.866	58.928	**<0.001**

*Abbreviations* OI: osteogenesis imperfecta; BSA: body surface area; SBP: systolic blood pressure; DBP: diastolic blood pressure; AD: aorta diameter; LAD: left atrium diameter; IVST: interventricular septum thickness; LVPWT: left ventricular posterior wall thickness; LVEDd: left ventricular end-diastolic diameter; LVESd: left ventricular end-systolic diameter; LVEF: left ventricular ejection fraction; FS: fractional shortening; LVM: Left ventricular mass. Bold *P* values indicate significance.

**Table 5:** The correlation between echocardiographic abnormalities and joint hypermobility in osteogenesis imperfecta

		Joint Hypermobility			
		Normal	Peripheral	Generalized	*P*
**A. In the juvenile group**					
Echocardiographic results	Normal	2	5	2	**0.004**
	abnormal	0	0	9	
**B. In the Adult group**					
Echocardiographic results	Normal	8	3	3	0.153
	Abnormal	0	5	1	

Bold *P* values indicate significance.

Simple regression analysis of echocardiographic parameters in juveniles showed that age, sex, BSA, SBP, and DBP were significant predictors of AD, LAD, IVST, LVPWT, LVEDd, LVESd, and LVM (except for IVST and LVPWT); OI, however, was the only significant predictor of LVPWT (Table 3A). In adults, sex and BSA were the significant predictors of AD, LAD, IVST, LVEDd, LVESd, and LVM (except sex for LAD and IVST) displayed by simple regression analysis. Age was the key predictor of AD, LAD, LVEF, and FS, whereas OI was an important predictor of AD and LVM (Table 3B).

Multiple regression analysis of echocardiographic parameters in juveniles showed that age and BSA were significant predictors of AD, IVST, and LVPWT among juveniles; sex and BSA were key predictors of LVEDd and LVESd; and BSA and OI were important predictors of LAD and FS (Table 4A). For adults, age was an important predictor of AD, LAD, FS, and LVM, whereas sex was the only significant predictor of LAD; BSA was a significant predictor of AD, LAD, LVPWT, LVEDd, LVESd, and LVM, and OI was a significant predictor of LAD and LVM (Table 4B).

### Joint hypermobility in OI and its correlation with cardiovascular abnormalities

Joint hypermobility was assessed in 18 of 25 juveniles with OI and two (11%) juveniles did not fulfill the criteria for a diagnosis of joint hypermobility, 6 (33%) had peripheral joint hypermobility, and 10 (56%) had generalized joint hypermobility. Among 23 OI adults, 20 underwent the joint hypermobility assessment: 8 (40%) adults did not have joint hypermobility, 8 (40%) had peripheral joint hypermobility, and 4 (20%) had generalized joint hypermobility.

In the OI juvenile group, the results of the Mann‒Whitney U test showed that the degree of joint hypermobility was significantly different between the echocardiographic normal and abnormal groups (*P* = 0.004) (Table 5A). Joint hypermobility had a wider impact on the echocardiographic abnormal group with OI. However, this was not found in the OI adult group (*P* = 0.153) (Table 5B).

## Discussion

In the present study, it should be noted that two groups of juveniles or adults were carefully matched by investigators so that they were comparable in age, sex, race, and blood pressure. As BSA has turned out to be an important factor in cardiac structural size, the ASE recommended correcting parameters by dividing by BSA [26]. By enrolling 25 juveniles and 23 adults with OI, we investigated the characteristics of cardiovascular abnormalities and joint hypermobility in Chinese patients with OI and analyzed the correlation between these two phenotypes in our cohort. Our results mainly suggest that mild valve regurgitation was the most common cardiovascular abnormality in both juveniles and adults with OI. Compared with healthy controls, some echocardiographic parameters, such as AD, LAD and IVST, were much larger and LVEF and LVM were significantly smaller in patients with OI only if corrected for BSA. In addition, joint hypermobility was more common in juveniles than in adults, and we also found that joint hypermobility had a wider impact on the echocardiographic abnormal group in juveniles with OI.

The cardiovascular connective tissues of the heart valves, aortic wall and heart chambers have a high concentration of type I collagen [9, 30]. Cardiac disorders are often associated with an accumulation, depletion, or restructuring of the collagen matrix [30]. Type I collagen is a heterotrimer composed of two α1 and one α2 chain that are assembled into a triple helix [4]. Most of the patients with OI result from structural or quantitative defects in the *COL1A1* or *COL1A2* genes that encode the α1 and α2 chains of collagen type I [2]. Preliminary experimental evidence based on OI mouse models has suggested that mutations in *COL1A1* or *COL1A2* will increase the risk of cardiovascular diseases. Weis and his colleagues revealed that [31] *Col1a2* mutation in the *oim* model produces significant alternations in the structural and mechanical properties of the ventricular myocardium, and a mild increase in LV wall thickness may represent compensation for reduced collagen. In addition, Pfeiffer et al. [32] researched the role of α2(I) collagen in aortic integrity in the *oim* model and suggested that the reduced thoracic aortic integrity was associated with the absence of the α2(I) collagen chains and a reduced collagen content. Based on the OI murine model *Aga2*, Thiele et al. [33] found that the downregulation of *Col1a1* transcripts caused a loss of extracellular matrix integrity and cardiopulmonary dysfunction.

Three previous BSA-matched studies [13, 34, 35] suggested that echocardiographic parameters were much larger (such as AD, LAD, IVST, LVEDd, and LVESd) or smaller (including LVEF and LVM) in OI than in controls. Another BSA-unmatched study [36] also revealed the same results, specifically when some parameters were indexed for BSA. The research mentioned above showed that LVEF or LVEF/BSA was much smaller in patients with OI, which means that cardiac function was impaired in OI. In our study, however, the corrected LVEF was larger in OI adults than in controls. A study in 58 children with OI found no cardiac abnormalities in type I patients, but cardiac alternations detected in type III [37]. Hence, we speculated the divergence of our study was attributed to the majority of our patients were clinical type 1, the insufficient sample size of our OI cohort, and the variations in the genetic backgrounds of different ethnic groups. Parameters such as AD, LAD, and IVST were significantly larger in patients with OI, implying compensation for reduced collagen, as verified by Weis [31]. Valvular regurgitation may occur when leaflets are thin, compliant, and slightly deformed [38]. Aortic root dilation and mitral regurgitation are the most commonly reported echocardiographic abnormalities in OI [39]. Radunovic et al. [36] studied the echocardiography of 99 adults with OI and found that 57.5% of patients had mild mitral regurgitation, 7.1% had moderate mitral regurgitation, 10.1% had mild aortic valve regurgitation, and 10.1% had moderate aortic valve regurgitation. In our study, mild mitral and tricuspid regurgitation were the most common abnormalities, especially the tricuspid regurgitation, whereas mild aortic valve regurgitation was only shown in OI adults. Two previous studies [14, 40] revealed that the mean arterial pressure (MAP) was much higher in OI groups than in controls. Radunovic et al. [36] also reported that approximately one-third of adult patients with OI had hypertension. Because of the lack of large-scale studies, however, there is still little understanding of hypertension prevalence in adults with OI. In our study, we excluded patients with hypertension as it may interfere with the assessment of echocardiography results. A 36-year-old female was the only patient in our study who had received cardiac surgery because of a congenital aortic valve defect when she was 25 years old. There was a lack of cardiac-related symptoms in her previous 25 years, and she even gave birth to a baby safely when she was 20 years old.

By multiple regression analysis in our study, age, sex and BSA were the main significant predictors of echocardiographic parameters, whereas OI was an important predictor only for LAD and FS in juveniles and for LAD and LVM in adults. Radunovic et al. [36] showed that SBP was also a significant predictor of some echocardiographic parameters in adults in multiple regression analysis. However, in our study, SBP and DBP showed significant impacts only in simple regression analysis. Zhao et al. [34] also researched the correlation between cardiovascular alterations and genotypes in OI through multiple regression analysis, and they showed that *COL1A1* mutation and defects in type I collagen were key predictors of cardiovascular abnormalities. They speculated that the difference of echocardiographic abnormalities might be correlated to the different changes in the structure and metabolism of type I collagen induced by various pathogenic gene mutations. In our study, most patients with echocardiographic abnormalities carried triple-helical structure change of type I collagen. However, we still need larger samples to test the effect of different genotypes on echocardiographic parameters.

Joint hypermobility is a shared feature of a group of genetic disorders affecting connective tissue matrix proteins, classically including EDS, Marfan syndrome, and OI [16]. Jansson et al. [41] revealed that females tend to be laxer than males and that the degrees of laxity decrease with age. In the European pediatric population, ligamentous laxity is considered to be present in 5–35% of children [42]. Arponen et al. [18] reported that joint hypermobility was found in 70% of pediatric OI patients, which was consistent with the research of Brizola et al. [43], who revealed joint hypermobility in 58–100% of pediatric OI patients. In our study, generalized joint hypermobility was 56% and 20% in OI juveniles and adults, respectively, which was similar to previous findings. Two patients in our study met the diagnostic criteria of C1ROD [22], who had blue sclera, generalized joint hypermobility, significantly soft and hyperextensible skin, atrophic scars and fracture history. The pathogenic variants were *COL1A1* (p. Arg1026*) and *COL1A2* (p. Cys1195Arg), respectively. In our study, we also investigated the correlation between cardiovascular abnormalities and the degree of joint hypermobility. Our results showed that joint hypermobility has a wider impact when echocardiography is abnormal in pediatric OI patients, which may suggest that type I collagen impairment is more severe when OI patients have abnormal cardiac manifestations, at the same time resulting in generalized ligament laxity. Radunovic et al. [36] showed that the same LV changes seem to be more pronounced in the OI type III group. A study of 58 OI children found no cardiac abnormalities in type I OI patients; however, cardiac alternations were detected in type III OI children [37]. In our study, among 9 juvenile patients with OI who had cardiovascular abnormalities, 6/9 patients were clinical type 3–4 (moderate to severe), which suggests that they had relatively more severe phenotypes. The results of these studies can strengthen our hypothesis. This finding also suggests that when doctors notice that a patient with OI who has prominent joint hypermobility, they should be alert for cardiovascular abnormalities. Larger samples and further research are needed to investigate the correlation.

Although preliminary studies and our research have demonstrated that most echocardiographic abnormalities are mild, asymptomatic and without the need for medical intervention, we still recommend that OI patients receive baseline echocardiography as early as possible. Like the female we mentioned above in our study who had a congenital aortic valve defect but not identified until her 25 years, even though it was lucky for her to be pregnant without accidents, as a doctor, we mustn’t gamble with patients’ luck. Joint hypermobility is a common extra-skeletal manifestation of OI, especially for juveniles, which may increase the risk of injury and fractures. On the other hand, there are some positive factors of hypermobility for artists and athletes that should be mentioned; for instance, dancers, violinists, pianists and gymnasts with lax finger and/or wrist joints can suffer less pain than their less flexible peers [44]. The female with the *IFITM5* mutation in our study said that she showed an advantage in playing the violin compared with her classmates when she was a young girl.

The samples in our study contained not only juveniles but also adults, which can add more real-world data to the relevant research. More importantly, we investigated the correlation between echocardiographic abnormalities and joint hypermobility in patients with OI for the first time. However, there are still some limitations to our study that must be considered. First, although we have studied the characteristics of echocardiography and joint hypermobility both in pediatric and adult OI patients, our study sample is not large enough to investigate the correlation between OI genotypes and echocardiographic parameters. Second, longitudinal data on echocardiography and the degree of joint hypermobility are scarce in our study, which prevents us from making concrete recommendations for cardiovascular surveillance. Third, due to the objective constraints of our study, we were unable to compare the degree of joint hypermobility between OI patients and controls.

## Conclusion

Our study mainly showed that mild valve regurgitation was the most common cardiovascular abnormality in both OI juveniles and OI adults. The differences in corrected echocardiographic parameters between OI and controls suggested that cardiac structure was damaged in OI. Joint hypermobility was more prevalent in juveniles than in adults. When echocardiography was abnormal, joint hypermobility had a wider impact on pediatric OI patients, which means that echocardiographic abnormalities may imply that the impairment of type I collage is more severe in OI. Although medical and surgical interventions were rarely needed in our study, we still recommend performing baseline echocardiographic assessments as early as possible among OI patients, and patients with cardiovascular abnormalities should be followed up intensively.

### Electronic supplementary material

Below is the link to the electronic supplementary material.

Supplementary Material 1 Characteristics of OI patients with echocardiographic abnormalities

## Data Availability

All data generated or analyzed during this study are included in the article, and further inquiries can be directed to the corresponding author.

## Abbreviations

OI: osteogenesis imperfecta
BMD: bone mineral density
EDS: Ehlers‒Danlos syndrome
C1ROD: *COL1*-related overlap disorder
SBP: systolic blood pressure
DBP: diastolic blood pressure
AD: aortic diameter
LAD: left atrium diameter
IVST: interventricular septum thickness
LVPWT: left ventricular posterior wall thickness
LVEDd: left ventricular end-diastolic diameter
LVESd: left ventricular end-systolic diameter
LVEF: left ventricular ejection fraction
FS: fractional shortening
LVM: Left ventricular mass
