# Supplementary material for: Echocardiographic abnormalities and joint hypermobility in Chinese patients with Osteogenesis imperfecta
Source: Orphanet J Rare Dis. 2024 Mar 12;19:116. doi: 10.1186/s13023-024-03089-x (PMC10935918; doi:10.1186/s13023-024-03089-x)
Supplement: Supplementary file 1 — Supplementary Material 1 Characteristics of OI patients with echocardiographic abnormalities [file 13023_2024_3089_MOESM1_ESM.docx]

**Supplementary Table 1** Characteristics of OI patients with echocardiographic abnormalities

|  | No | Sex | Age (ys) | BSA (m^2^) | SBP/DBP (mmHg) | Clinical type | Cardiac-related symptoms | Echocardiographic abnormality | Ligamentous laxity | Hemosiderotic  scarring | Mutation gene |
| --- | --- | --- | --- | --- | --- | --- | --- | --- | --- | --- | --- |
| Juveniles | 1 | F | 5 | 0.71 | 89/56 | 1 | Asymptomatic | Mild tricuspid regurgitation | Generalized | No | *COL1A2*, c.847G>A |
|  | 2 | F | 9 | 1.26 | 98/63 | 4 | Asymptomatic | Mild tricuspid regurgitation | Generalized | No | *COL1A1*, c.1148G>C |
|  | 3 | M | 9.5 | 1.22 | N.A. | 3 | Asymptomatic | Mild mitral and tricuspid regurgitation | Generalized | No | WNT1, c.500dupG/c.506G>A |
|  | 4 | M | 11 | 1.16 | N.A. | 4 | Asymptomatic | Mild tricuspid regurgitation | Generalized | No | *COL1A1*, c.3540delC |
|  | 5 | M | 12.3 | 1.22 | 106/53 | 4 | Asymptomatic | Mild tricuspid regurgitation | Generalized | No | *COL1A2*, c.2314G>A |
|  | 6 | M | 13.4 | 2.01 | N.A. | 4 | Asymptomatic | Mild tricuspid regurgitation | Generalized | No | *COL1A1*, c.3655G>A |
|  | 7 | M | 13.9 | 1.42 | 110/76 | 4 | Asymptomatic | Mild tricuspid regurgitation | Generalized | No | *COL1A2*, c.1009G>A |
|  | 8 | M | 14 | 1.65 | 116/78 | 1 | Asymptomatic | Mild mitral and tricuspid regurgitation | Generalized | No | *COL1A1*, c.769G>A |
|  | 9 | F | 17 | 1.55 | 133/86 | 1 | Asymptomatic | Mild mitral and tricuspid regurgitation | Generalized | Yes | *COL1A2*, c.3583T>C |
| Adults | 10 | F | 29 | 1.36 | 107/73 | 1 | Asymptomatic | Mild tricuspid regurgitation | Peripheral | Yes | *COL1A1*, c.3421C>T |
|  | 11 | F | 36 | 1.54 | 103/70 | 1 | syncope | Aortic valve replacement history | Peripheral | No | *COL1A2*, c.268G>T |
|  | 12 | F | 41 | 1.33 | N.A. | 1 | Asymptomatic | Mild mitral regurgitation | Peripheral | No | *COL1A1*, c.3235G>A |
|  | 13 | F | 42 | 1.48 | 120/77 | 1 | Asymptomatic | Mild mitral regurgitation | Peripheral | Yes | *COL1A1*, c.588+2T>A |
|  | 14 | F | 50 | 1.35 | 111/74 | 1 | Palpitation | Mild mitral and tricuspid regurgitation, left atrium enlargement, and increased pulmonary artery pressure | N.A. | No | *COL1A1*, c.1703delC |
|  | 15 | F | 58 | 1.36 | 131/83 | 1 | Asymptomatic | Mild tricuspid regurgitation | N.A. | No | *COL1A2*, c.1009G>A |
|  | 16 | F | 62 | 1.67 | 104/71 | 1 | Asymptomatic | Mild tricuspid regurgitation | Peripheral | No | *COL1A1*, c.268G>T |
|  | 17 | F | 63 | 1.44 | 126/68 | 1 | Asymptomatic | Mild aortic regurgitation | Peripheral | No | *COL1A1*, c.268G>T |

OI: osteogenesis imperfecta; BSA: body surface area; SBP: systolic blood pressure; DBP: diastolic blood pressure; N.A. : not available.
